# The volume of brisk walking is the key determinant of BMD improvement in premenopausal women

**DOI:** 10.1371/journal.pone.0265250

**Published:** 2022-03-16

**Authors:** Yong-Sheng Lan, Yu-Juan Feng

**Affiliations:** 1 School of Physical Education, Changchun Normal University, Changchun, Jilin, China; 2 Shandong University of Art and Design, Jinan, Shandong, China; Nanjing Medical University, CHINA

## Abstract

**Summary:**

Osteoporosis is an increasing health problem in postmenopausal women. Our findings indicated that long-term brisk walking with a volume greater than 16 per week is effective for improving BMD in premenopausal women.

**Purpose:**

To examine the effects of brisk walking on bone mineral density (BMD) in premenopausal women, and further determine the effective frequency, intensity, time and volume (frequency x duration) of brisk walking for training strategy prescription.

**Methods:**

222 healthy premenopausal women were recruited for BMD measurement. According to the survey of their physical activity level, 84 subjects (age: 46±1.8) whose physical activity index ≥40 were categorized into the brisk walking group, and 138 subjects (age: 47±2.2) whose physical activity index <40 were assigned to the sedentary group. The BMD of these two groups were statistically compared with an independent t test. Next, 35 subjects from the original sedentary group were recruited for BMD measurement after 2-year moderate brisk walking. According to the volume of physical activity per week, they were divided into the control group (n = 10, aged 49±0.9), volume 8 group (n = 4, aged 48±1.2), volume 12 group (n = 7, aged 49±1.4), volume 16 group (n = 8, aged 49±1.3), and volume 20 group (n = 6, aged 49±1.5). ANOVA was used to analyze BMD before and after brisk walking among the five groups.

**Results:**

The BMD in the brisk walking group (1.00±0.008 g/cm^2^) was significantly higher than that in the sedentary group (0.89±0.008 g/cm^2^) (P<0.001). Stepwise regression analysis revealed that the volume of brisk walking was significantly correlated with BMD (P<0.001). In particular, brisk walking with a volume greater than 16 (a score of duration up to 4 and a score of frequency up to 4 or 5) per week is effective for improving BMD in premenopausal women (P = 0.03, P = 0.002, respectively).

**Conclusions:**

Long-term brisk walking is an efficient way to improve BMD. Taking brisk walks for 30 minutes per day 3 or more times per week (volume>16) is recommended to prevent bone loss in premenopausal women.

## Introduction

Osteoporosis is an increasing health problem in postmenopausal women whose hormonal changes lead to rapid bone loss [1]. Pharmaceutical agents [2–5], high dietary calcium intake [6, 7] and physical activity [8, 9] have been validated to increase bone mineral density (BMD) and prevent bone loss. From the perspective of prevention, physical activity is accepted as an optimal intervention due to its extensive benefits on multiple systems in addition to bone, such as muscle [10], the cardiovascular system [11] and the nervous system [12], as well as the coeffects of multiple systems on the bone.

Walking is a rhythmic, dynamic, aerobic activity. It strengths the major muscle groups of the legs, limb girdle and lower trunk, as well as muscles of the shoulder girdle, increases the flexibility and stability of cardinal joints, improves cardiovascular and respiratory functions, and promotes energy expenditure and weight control [13]. Walking confers multifarious benefits to physical fitness with minimal adverse effects. Brisk walking is a moderate-intensity aerobic exercise. As defined by the Centers for Disease Control and Prevention (CDC), brisk walking entails maintaining a walking pace of at least 3.0 miles per hour or 20 minutes per mile. Besides of the benefits mentioned above, brisk walking was suggested to stimulate bone turnover [14, 15] and improve calcaneal and lumbar BMD [16, 17]. However, this positive effect of brisk walking on bone is disproved by some other research. For example, a 52-week walking program reported by Cavanaugh and Cann did not prevent the loss of spinal trabecular bone density in early-postmenopausal women [18]; a 10-week walking intervention was reported to be insufficient for the improvement of distal forearm and calcaneus BMD [19]. To date, it is still not clear if brisk walking is capable of preventing bone loss or increasing BMD. To clarify this problem, it is much more necessary to determine the effective frequency, intensity and time for the prescription of brisk walking strategies.

So, the aim of this study was to make clear the effect of brisk walking on BMD improvement in premenopausal women and find out the key factor for improving BMD.

## Methods

### Participants

The project starts from 2015 and ends at 2018. Healthy premenopausal women aged 45 to 50, working at universities, colleges or government departments, living in Changchun, Jilin Province in China were recruited at the same time period via advertising (including brochures, fliers and posters). They were all office workers and mainly engaged in mental work. All subjects volunteered to participate in the study. Written informed consent were obtained from all the subjects.

#### Inclusion criteria

222 subjects were enrolled according to the following criteria in 2015: premenopausal women, aged 45 to 50, willingness to participate, clinically healthy (no diabetes; no cardiovascular, musculoskeletal or respiratory diseases; and no psychiatric diseases), no dieting, nonsmoking, no use of medication or health care products known to affect the skeleton, and no regular exercise activities except for brisk walking.

#### Physical activity assessment

The levels of physical activity (PA), including frequency, intensity, duration and physical activity index (frequency x intensity x duration), were assessed using Physical Activity Index questionnaire according to John C. Griffin (Griffin, 1998) (S1 Table). The Physical Activity Index questionnaire was a self-reported questionnaire and had been validated by previous studies [20]. It asked the subjects about their physical activities of the last 6 months. Moreover, the physical activity volume (frequency x duration) was also assessed.

As defined by the Physical Activity Index questionnaire that people whose physical activity index ≥40 could be considered to take part in sports regularly. In this study, 84 subjects whose physical activity index was ≥40 were categorized into the brisk walking group. A total of 138 subjects whose physical activity index was <40 were assigned to the sedentary group. These two groups were compared to analyze whether brisk walking had positive effects on BMD.

#### Cohort study

138 subjects in the sedentary group were told the positive effects of brisk walking on BMD and verbally advised to carry out brisk walking at a moderately heavy intensity level, as in cycling and other recreational sports (intensity score = 3) in 2016. They were not monitored or contacted in any way during the 2-years follow-up period.

**Fig 1:**
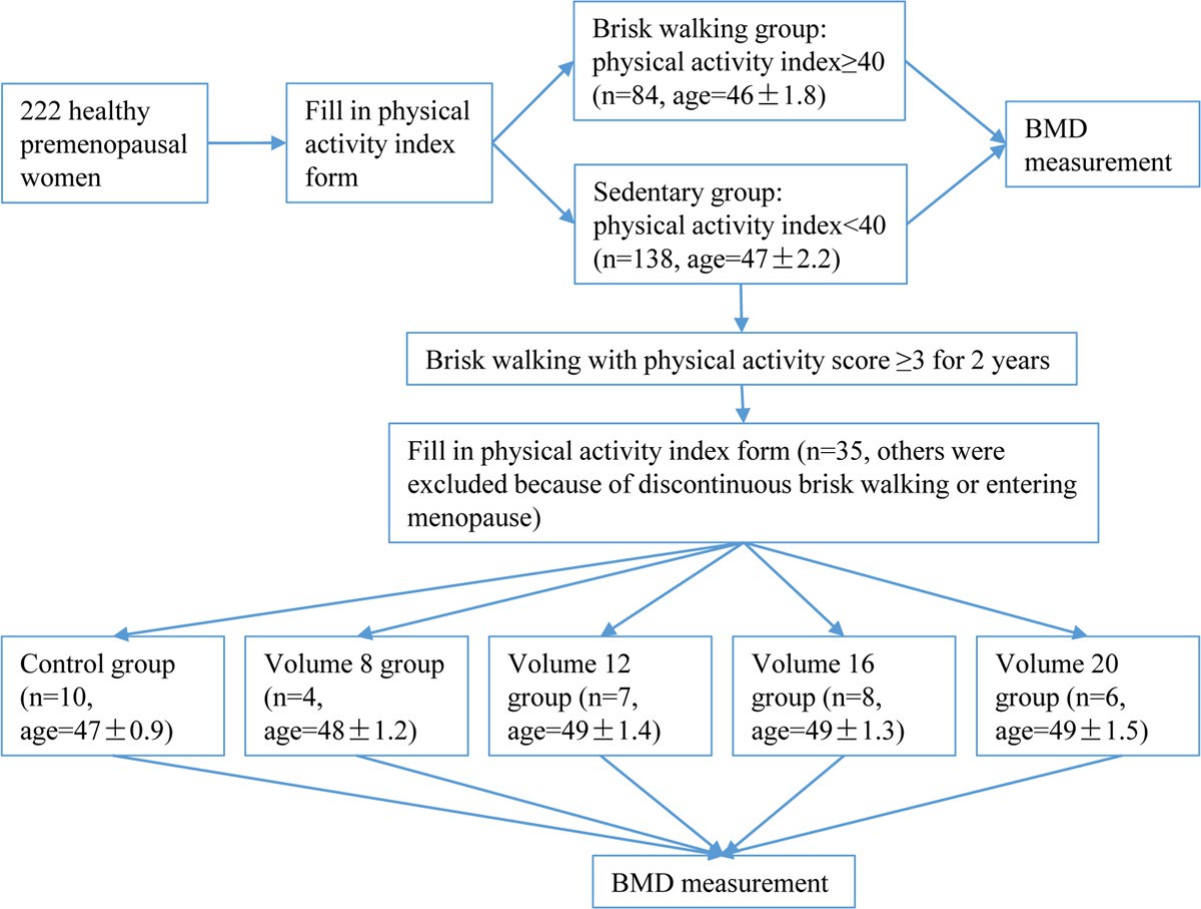
Outline of the study.

Physical activities were assessed after 2 years. According to retrospective self-reports, 35 subjects were enrolled in the cohort study while the other 103 subjects were excluded because they performed discontinuous and erratic brisk walking, or entering menopause. According to the volume of physical activity per week, the 35 subjects were divided into the control group, volume 8 group, volume 12 group, volume 16 group, and volume 20 group. They were asked to measure BMD in our lab again. The design of the study was illustrated in Fig 1. The whole project lasted for 3 years.

The protocols and procedures used in this study were approved by the Changchun Normal University’s Ethical Review Board. The methods were carried out in accordance with the approved guidelines.

#### Bone densitometry

Bone mineral densitometry measurements were performed by trained personnel at Changchun Normal University using dual energy X-ray absorptiometry (Norland XR-800). With the subjects lying in a supine position on a padded table, an X-ray beam passes from head to feet direction through the whole body. In details, the whole body scan requires the operator to mark the start and baseline points. Have the patient lie on the table, face up with the head oriented to the right side of the table, (operator facing the table) and without pillow under the patient’s head. Marking the start point: Turn on the laser. Position the laser dots 1 cm above the top of the center of the patient’s head, and press the mark button on the scanner arm touch pad. Marking the baseline point: Move the scanner arm over the patients’ abdomen. Turn on the laser. Position the laser dot at a point on the abdomen adjacent to the spine and midway between the lowest rib and the iliac crest. Mark in an area of maximum soft tissue and no bone. Press the mark button. Position the top edge of the chest cursor to just under the chin. Position the upper control points above the junctions of the humerus and scapula. Position the bottom control points to include the rib cage. Position the pelvic cursor to encompass the pelvis, yet containing a minimum of midriff, leg, and femoral neck tissue. Position the leg cursors so that both legs are encompassed. Intelligent scanning (i.e. scanning body edge to body edge) is used to minimize scan time and to ensure the scan automatically stops after scanning the patient’s feet. The BMD of the subwhole body regions was measured. The effective radiation dose was <0.2 mSv per scan (Whole-body).

Data analyses were performed using Illuminatus DXA software (Norland Corporation, Oslo, Norway) after the scan was done. And the reference of the whole body scan was performed as follows: Position the upper edge of the chest cursor just below the chin. Position the upper control point over the junction of the humerus and scapula. Position the bottom control point between the arm and torso, including the rib cage. Position the pelvic cursor to encompass the pelvis, but include a minimum of mid-lumbar, leg, and femoral neck tissue. Position the upper control point between the arms and torso. Position the leg cursors so that both legs are included, with the center line separating the legs. Reference database at whole body for Chinese females was selected in this study.

To ensure high quality results, a daily system quality assurance calibration is required. To calculate precision error, four volunteers underwent three repeated whole body BMD measurement with repositioning to assess repeatability between studies. The results of the three studies were averaged for individual individuals during the analysis to calculate the coefficient of variation. The coefficient of variation in our laboratory for the whole body BMD is 1.25%.

### Statistical analyses

SPSS 17.0 was used for analyses. Descriptive statistics was used to test the normal distribution. An independent t test was used to compare the differences in BMD between the sedentary women and the brisk-walking women. The correlation analysis was used to detect the correlations between BMD and brisk walking factors. Stepwise regression was used to reveal which factors of brisk walking had the greatest impact on BMD in premenopausal women. ANOVA was used to test the BMD before and after brisk walking among the control group, volume 8 group, volume 12 group, volume 16 group, and volume 20 group.

## Results

To ensure that brisk walking has positive effects on BMD in premenopausal women, 222 subjects were divided into a brisk walking group whose physical activity index was ≥40 (n = 84, aged 46±1.8) and a sedentary group whose physical activity index was <40 (n = 138, aged 47±2.2). Kolmogorov-Smirnov value in the test of normality was great than 0.05. Then independent t test was used to compare the differences in BMD between those two groups. We found that the BMD of the women in the brisk walking group was significantly higher than that of the women in the sedentary group (Table 1, P<0.001). Besides, the weight and Body Mass Index (BMI) of participants were also analyzed and there were no significant differences between those two groups (Table 1). It was demonstrated that brisk walking improves BMD in premenopausal women.

**Table 1:** Comparison of BMD between sedentary group and brisk walking group.

Groups	n	BMD (g/cm^2^, x±SE)	Weight (kg, x±SE)	BMI (kg/m^2^, x±SE)
Sedentary	138	0.89±0.008***	66.7±1.13	26.57±0.44
Brisk Walking	84	1.00±0.008	68.86±2.15	26.82±0.23

*** P<0.001

Next, we aimed to investigate which factor of brisk walking had the greatest impact on BMD in premenopausal women. We first used correlation analysis to detect the correlations between BMD and brisk walking factors in all 222 subjects. We found that the intensity, duration, frequency, volume and physical activity index of brisk walking had significant correlations with BMD (Table 2, R = 0.37, P<0.001; R = 0.60, P<0.001; R = 0.65, P<0.001; R = 0.70, P<0.001; R = 0.62, P<0.001; respectively). However, it was difficult to conclude whether the intensity, duration, frequency, volume and physical activity index of brisk walking all had important impacts on BMD. Because these five factors were significantly correlated with each other (Table 3). In particular, the R value between volume and frequency was as high as 0.97. In this case, the volume and frequency may be considered one factor. Therefore, we further used the stepwise regression analysis and found that only the volume of brisk walking was significantly correlated with BMD (Table 4, P<0.001). It is suggested that brisk walking volume is the key factor impacting BMD in premenopausal women.

**Table 2:** Correlations between BMD and intensity, duration, frequency, volume and index.

		Intensity	Duration	Frequency	Volume	Index
BMD	R	0.37	0.60	0.65	0.70	0.62
	P	<0.001	<0.001	<0.001	<0.001	<0.001

**Table 3:** Correlations between intensity, duration, frequency, volume and index.

		Intensity	Duration	Frequency	Volume	Index
Intensity	R		0.70	0.67	0.59	0.76
	P		<0.001	<0.001	<0.001	<0.001
Duration	R	0.70		0.89	0.91	0.79
	P	<0.001		<0.001	<0.001	<0.001
Frequency	R	0.67	0.89		0.97	0.86
	P	<0.001	<0.001		<0.001	<0.001
Volume	R	0.59	0.91	0.97		0.89
	P	<0.001	<0.001	<0.001		<0.001
Index	R	0.76	0.79	0.86	0.89	
	P	<0.001	<0.001	<0.001	<0.001	

**Table 4:** Stepwise regression between BMD and intensity, duration, frequency, volume and index.

		Intensity	Duration	Frequency	Volume	Index
BMD	P	0.655	0.179	0.067	<0.001	0.215

**Fig 2:**
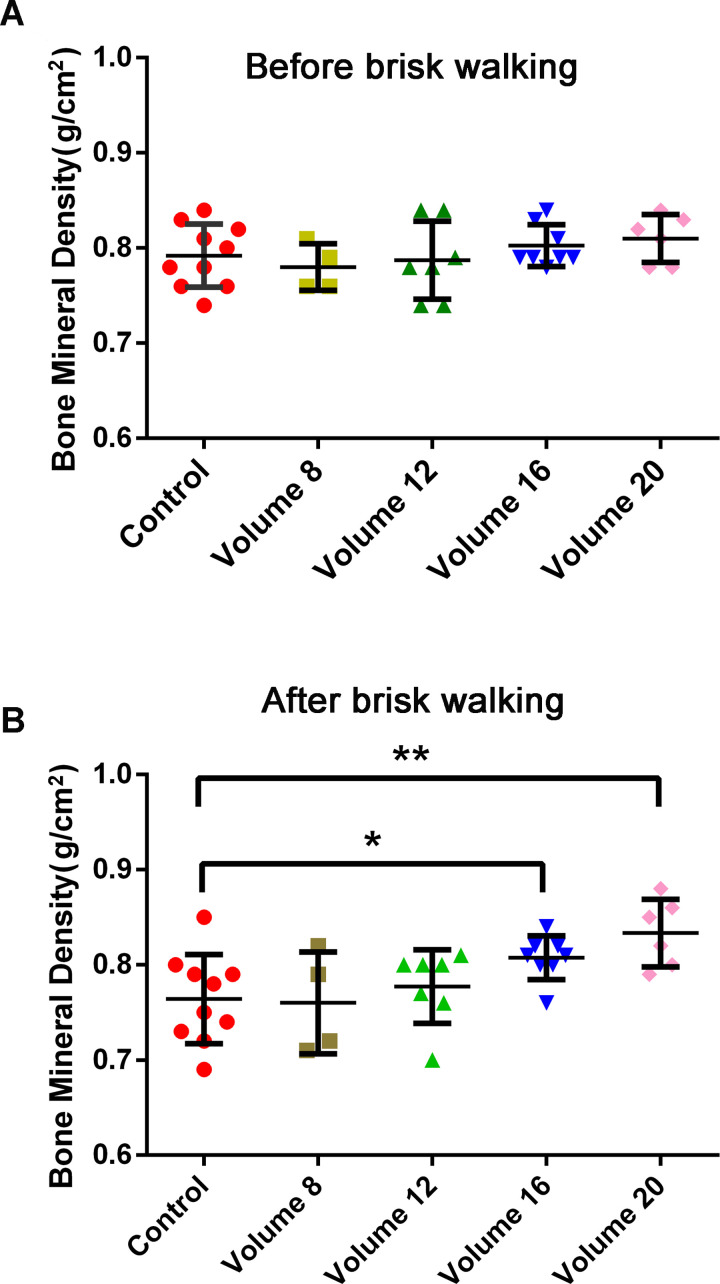
Comparison of BMD between the control group, the volume 8 group, the volume 12 group, the volume 16 group and the volume 20 group before and after brisk walking. (A) There were no significant differences between the control group, the volume 8 group, the volume 12 group, the volume 16 group and the volume 20 group before brisk walking. Volume = frequency x duration, which was calculated according to the self-reported Physical Activity Index questionnaire. The subjects whose volume was ≈ 8 were assigned to the volume 8 group and so forth. (B) Compared with the control group, significant differences were observed in the volume 16 group and the volume 20 group. *P<0.05; **P<0.01.

To further explore how much volume was effective, we asked all of the women in the sedentary group to take up brisk walking with the following protocol: Intensity: Physical activity score ≥3 (moderately heavy); Frequency: No requirements; and Duration: No requirements. After two years, they were invited to get their BMD measured and were asked to fill in the physical activity index form again. People who had entered menopause were excluded from the statistics. According to the information that was provided on the physical activity index form, people whose physical activity index score was <40 were assigned to the control group (n = 10, aged 49±0.9); people whose volume was ≈ 8 were assigned to the volume 8 group (n = 4, aged 48±1.2); people whose volume was ≈ 12 were assigned to the volume 12 group (n = 7, aged 49±1.4); people whose volume was ≈ 16 were assigned to the volume 16 group (n = 8, aged 49±1.3); and people whose volume was ≈ 20 were assigned to the volume 20 group (n = 6, aged 49±1.5). Normality distribution test was conducted both before and after brisk walking, Kolmogorov-Smirnov values were great than 0.05. Then ANOVA was used to analyze BMD before and after brisk walking among the five groups. The BMD showed no significant difference among the five groups before brisk walking (Fig 2A). After brisk walking, the BMD of the volume 16 group and volume 20 group were significantly higher than that of the control group (Fig 2B, P = 0.03, P = 0.002). No significant differences were found in the volume 8 group and the volume 12 group. Besides, the weight and BMI showed no significant differences among the five groups before and after 2-years brisk walking (S2 and S3 Tables). These results demonstrated that brisk walking at a volume > = 16 was more effective.

Taken together, long-term brisk walking was an efficient way to improve BMD in premenopausal women. Notably, volume (frequency x duration) of brisk walking was the key factor impacting BMD. Taking brisk walks with volume>16 (30 minutes per day, 3 or more times per week) was recommended to prevent bone loss in premenopausal women.

## Discussion

Osteoporosis, a disease characterized by low BMD, can increase fracture risk, reduce functionality, and cause disability and even death [21]. Physical activity is a good way to improve or prevent osteoporosis. One of the key strategies for preventing osteoporosis relies on maximizing the peak BMD. Since 40% of adult BMD accumulates during pubertal years [22], adolescence is a critical stage to increase baseline BMD. However, several reviews have shown that physical activity has consistent positive effects on bone development, and thus, special physical activity recommendations for adult individuals are proposed.

Brisk walking is a sustained dynamic aerobic exercise, which, similar as other forms of aerobic exercise, e.g. swimming and cycling, improves the heart, lungs and cardiovascular function. It is common to people of all age, particularly prevalent to the middle-aged and the elderly. No special skills or equipment are required. Brisk walkers can self-regulate the intensity, duration and frequency. Compared with jogging or running, brisk walking has a low ground impact, minimizing the strain on feet and joints and is relatively risk-free. Besides, there is little, if any, decline in middle age. Totally, it is the main option for increasing physical fitness in sedentary populations, especially in the middle-aged and the elderly.

Strength exercise, which is mainly performed in the manner of rapid jumping, is considered to be the most efficient way to improve BMD, especially in children [23]. However, the effects of aerobic exercise on BMD improvement are controversial. Some people found that Tai-chi improved BMD [24], while others found that swimming and marathon running had no effect on BMD improvement [25, 26]. This inconsistency may be caused by the different exercise types. In our study, we focused on brisk walking and found that premenopausal women who usually take brisk walks have a higher BMD than others who live a stationary life (Table 1). It is suggested that brisk walking has positive effects on BMD in premenopausal women. But some early studies reported that there were no effects of brisk walking on BMD [18, 19]. We think the controversy is mainly caused by the short intervention period. The intervention period of those studies was 52 weeks and 10 weeks, respectively. However, bone metabolism is a slow process, especially in mid-aged and elderly people. Some studies have noted that it can take 12 months, or longer in people with osteoporosis, of bone remodeling before a new steady state of bone metabolism can be reached [27]. Depending on osteoporosis status, we think more than 1 year may be needed to study the effects of brisk walking on BMD. According to our study, we found that 2 years of brisk walking has positive effects on BMD.

The parameters of physical activity include type, frequency, intensity, duration, volume, and physical activity index. All of these factors are important for the effects of physical activity [28]. As observed in our study, the frequency, intensity, duration, volume and physical activity index of brisk walking were all strongly associated with BMD (Table 2). We also found that these five factors were highly associated with each other (Table 3). To identify the key factor of brisk walking that affects BMD, a stepwise regression analysis was performed. The results showed that only volume was strongly associated with BMD improvement (Table 4), which suggested that volume was the dominant factor affecting BMD. Next, to further verify the speculation, we asked people whose physical activity index was <40 to take brisk walks with an intensity score >3 but without a specified requirement for duration or frequency. The BMD of the participants was measured again 2 years later. The results showed that brisk walking with a volume greater than 16 per week can effectively prevent the loss of BMD, while a volume of less than 16 per week is ineffective (Fig 2B). This finding demonstrated that the volume of brisk walking played a key role in preventing bone loss. There was a beneficial effect on BMD only when the score of duration reached 4 and the score of frequency reached 4 or 5. In other words, over 30 minutes of brisk walking more than 3 times per week is effective.

There are two limitations in this study. First, the body composition of volunteers in our experiment, such as muscle weight, fat weight and body fat percentage (BFP) were not collected, so it remains unclear whether the positive effects of brisk walking on premenopausal women were caused by brisk walking directly and/or by brisk walking-mediated body composition change. Second, the spine and hip are more prone to osteoporosis fracture, thus BMD at the lumbar spine and femoral neck are usually measured to diagnose osteoporosis or low bone mass [29]. In this study, we measured the BMD of the whole body, which was unable to show the BMD of the above common sites for osteoporotic fracture.

In conclusion, we demonstrate that long-term brisk walking is an effective way to improve or slow the downward trend in BMD in premenopausal women. Taking brisk walks for 30 minutes per day 3 or more times per week (volume>16) is recommended to prevent bone loss.

## Supporting information

S1 Table Questionnaire for physical activity index. (DOCX)

S2 Table Comparison of weight and BMI between difference groups before 2-years brisk walking. (DOCX)

S3 Table Comparison of weight and BMI between difference groups after 2-years brisk walking. (DOCX)

S1 Raw data Raw data behind Table 1. (XLSX)

S2 Raw data Raw data behind Tables 2–4. (XLSX)

S3 Raw data Raw data behind Fig 2. (XLSX)

S4 Raw data Raw data behind S2 and S3 Tables. (XLSX)

## Abbreviations

ANOVA: analysis of variance
BMD: bone mineral density
CDC: Centers for Disease Control and Prevention
