# Supplementary material for: The volume of brisk walking is the key determinant of BMD improvement in premenopausal women
Source: PLoS One. 2022 Mar 16;17(3):e0265250. doi: 10.1371/journal.pone.0265250 (PMC8926180; doi:10.1371/journal.pone.0265250)
Supplement: S1 Table — (DOCX) [file pone.0265250.s001.docx]

**Physical Activity Index**

Evaluate your current exercise program by selecting your score for each category.

|  | **Score** | **Activity** |
| --- | --- | --- |
| **Intensity** |  |  |
|  | 5 | Sustained heavy breathing and perspiration |
|  | 4 | Intermittent heavy breathing and perspiration, as in tennis |
|  | 3 | Moderately heavy, as in cycling and other recreational sports |
|  | 2 | Moderate, as in volleyball, softball |
|  | 1 | Light, as in fishing |
| **Duration** |  |  |
|  | 4 | Over 30 minutes |
|  | 3 | 20 to 30 minutes |
|  | 2 | 10 to 20 minutes |
|  | 1 | Less than 10 minutes |
| **Frequency** |  |  |
|  | 5 | 6 to 7 times per week |
|  | 4 | 3 to 5 times per week |
|  | 3 | 1 to 2 times per week |
|  | 2 | A few times per month |
|  | 1 | Less than once a month |

Intensity X Duration X Frequency = Score Total

Your Score: x x =

| **Evaluation of Activity Score** | | |
| --- | --- | --- |
| **Score** | **Evaluation** | **Activity Category** |
| 81 to 100 | Very active lifestyle | High |
| 60 to 80 | Active and healthy | Very good |
| 40 to 59 | Acceptable but could be better | Fair |
| 20 to 39 | Not good enough | Poor |
| Under 20 | Sedentary |  |
