# Supplementary material for: The volume of brisk walking is the key determinant of BMD improvement in premenopausal women
Source: PLoS One. 2022 Mar 16;17(3):e0265250. doi: 10.1371/journal.pone.0265250 (PMC8926180; doi:10.1371/journal.pone.0265250)
Supplement: S2 Table — (DOCX) [file pone.0265250.s002.docx]

Supplement Table 2-1 Comparison of weight and BMI between difference groups before 2-years brisk walking

|  | n | Weight (kg, x±SE) | BMI (kg/m^2^, x±SE) |
| --- | --- | --- | --- |
| Control | 10 | 60.4±2.21 | 25.2±0.88 |
| Volume 8 | 4 | 61.75±2.56 | 25.5±1.32 |
| Volume 12 | 7 | 61.86±3.97 | 24.29±1.51 |
| Volume 16 | 8 | 62.88±3.23 | 24.38±1.07 |
| Volume 20 | 6 | 64.5±3.47 | 24.33±1.05 |
