# Supplementary material for: The volume of brisk walking is the key determinant of BMD improvement in premenopausal women
Source: PLoS One. 2022 Mar 16;17(3):e0265250. doi: 10.1371/journal.pone.0265250 (PMC8926180; doi:10.1371/journal.pone.0265250)
Supplement: S3 Table — (DOCX) [file pone.0265250.s003.docx]

Supplement Table 2-2 Comparison of weight and BMI between difference groups after 2-years brisk walking

|  | n | Weight (kg, x±SE) | BMI (kg/m^2^, x±SE) |
| --- | --- | --- | --- |
| Control | 10 | 59.7±2.17 | 24.9±0.75 |
| Volume 8 | 4 | 64±3.19 | 26.75±1.93 |
| Volume 12 | 7 | 61.86±4.23 | 25±1.62 |
| Volume 16 | 8 | 63.13±3.81 | 24.38±1.34 |
| Volume 20 | 6 | 67.33±4.19 | 25.5±1.23 |
